# Numerical Simulation of Particle Dynamics in a Spiral Jet Mill via Coupled CFD-DEM

**DOI:** 10.3390/pharmaceutics13070937

**Published:** 2021-06-23

**Authors:** Satyajeet Bhonsale, Lewis Scott, Mojtaba Ghadiri, Jan Van Impe

**Affiliations:** 1 BioTeC+, Department of Chemical Engineering, Technology Campus Ghent, KU Leuven, Gebroeders de Smetstraat 1, 9000 Ghent, Belgium; satyajeetsheetal.bhonsale@kuleuven.be; 2 School of Chemical and Process Engineering, University of Leeds, Leeds LS2 9JT, UK; pm11lms@leeds.ac.uk (L.S.); M.Ghadiri@leeds.ac.uk (M.G.)

**Keywords:** spiral jet mills, discrete element models, computational fluid dynamics

## Abstract

Spiral jet mills are ubiquitous in the pharmaceutical industry. Breakage and classification in spiral jet mills occur due to complex interactions between the fluid and the solid phases. The study of these interactions requires the use of computational fluid dynamics (CFD) for the fluid phase coupled with discrete element models (DEM) for the particle phase. In this study, we investigate particle dynamics in a 50-mm spiral jet mill through coupled CFD-DEM simulations. The simulations showed that the fluid was significantly decelerated by the presence of the particles in the milling chamber. Furthermore, we study the particle dynamics and collision statistics at two different operating conditions and three different particle loadings. As expected, the particle velocity was affected by both the particle loading and operating pressure. The particles moved slower at low pressures and high loadings. We also found that particle–particle collisions outnumbered particle–wall collisions.

## 1. Introduction

Particle size reduction is an important step in the design, development, and processing of active pharmaceutical ingredients (API). Spiral jet mills are the preferred comminution devices for ultra-fine grinding where particles less than 10 µm diameter are desired [1,2]. Since spiral jet mills were first patented in the 1930s [3], their design has remained relatively unchanged. Their design consists of a short cylindrical (or elliptical) milling chamber into which high velocity gas is pushed through several nozzles (called the grinding nozzles), which are at an angle to the mill perimeter. The gas jets entering through these nozzles create a vortex in the milling chamber.

Solid feed particles are fed to an injector, which delivers the feed to the vortex, wherein they are accelerated by the gas flow. The momentum gathered by the particles due to the high velocity gas jets leads to high energy particle–particle and particle–wall collisions, which cause breakage. The centrifugal forces in the vortex retain the coarse particles within the milling chamber. The centrifugal forces and the radial drag forces acting on the particles in the vortex depend on the particle size (*x*). As the size decreases due to breakage, centrifugal force (∼$x^{3}$) reduces faster than the radial drag forces (∼$x^{2}$).

When the radial drag force acting on a particle exceeds the centrifugal force, the particle is entrained out of the milling chamber via an outlet in the centre of the mill. Although the energy consumption of spiral jet mills is relatively high, they provide various advantages. Due to the absence of any moving parts and the self-classifying nature of the mill, contamination can be completely avoided [4]. Moreover, the expansion of fluid from the jet into the grinding chamber leads to a cooling effect, which makes spiral jet mills attractive for heat sensitive materials [5].

The popularity of the jet mills arises from the simplicity of its operation. In general, only three parameters are needed to control the operation of the jet mills: the injector nozzle pressure (IP), the grinding nozzle pressure (GP), and the solid feed rate (FR). Of these, the GP and FR have a significant impact on the milling performance, while the impact of IP is not large [6]. The kinetic energy within the jet mill is directly related to the GP. Higher kinetic energy leads to enhanced acceleration of the particles and hence higher impact collisions.

A large number of studies assessing the influence of the GP (or in some cases the gas flow rate) on milling performance arrived at the same conclusion: increasing the GP leads to a decrease in the output median particle size [7,8,9,10,11]. As the IP has a negligible impact on the mill performance, it is usually maintained at a pressure that is slightly higher than the GP. Increasing the FR makes the output particle size coarser. Although the frequency of collisions increases with increasing FR [12], the high particle concentration within the mill causes the fluid energy in the mill to dissipate faster [13]. This leads to low energy collisions.

A systematic mathematical description of the spiral jet mill is desirable for predictive purposes. A validated model can reduce the need for extensive experimentation with an expensive API. Moreover, a model-based process understanding is also a key element of the Quality by Design paradigm adopted by the Food and Drugs Authority in the United States. Despite the prevalence of spiral jet mills in the pharmaceutical industry, modelling studies are relatively sparse. Some of these studies rely on the force and energy balance approach [14,15,16], some on the population balance approach [17,18,19], and a few on computational fluid dynamics [15,20,21].

The energy and force balances equate the two opposing forces acting on the particles: the centrifugal force and the radial drag force. Based on the balance, a particle cut size that depends on the ratio of tangential and radial particle velocities (called the spin number) is derived. Under idealistic assumptions of Archimedes spiral flow, Tanaka [16] derived expressions that defined the tangential and radial velocity in a mill as a function of the mill parameters. Rodnianski et al. [15], on the other hand, used CFD simulations to obtain the spin number and radial velocity. They described the spin number as a general function of mill’s geometric and operational parameters. An important conclusion from the CFD analysis of Rodnianski et al. [15] is that the gas flow rate does not affect the spin number. MacDonald et al. [14] built upon the previous results and derived a cut size equation by incorporating the energy balance. In all the above derivations, the particle tangential velocity was assumed to be the same as the gas velocity.

Population balance modelling (PBM) has also been used to describe breakage is described with empirical breakage distribution functions. As the fully described PBM is a complex integro-differential set of equations, numerical methods are commonly utilized to obtain a solution [22]. Gommeren et al. [17] presented a compartmentalized PBM describing three zones within the grinding chamber: the comminution zone, central (feed) zone, and the classifier zone. The model was then used to determine the residence time distribution, hold up, and closed-loop control. However, no discussion on the estimation of the parameters involved in the model was provided.

An highly empirical steady-state PBM was also described in Starkey et al. [18]. However, the algebraic equation set only considered six discrete size classes to describe the particle size distribution of the product. A major drawback in the PBM approach is the need for estimating the breakage parameters from experimental data. Although a variety of approaches have been proposed for this inverse problem, they cannot be applied directly to the spiral jet mill models. Bhonsale et al. [23] performed an identifiability analysis of a discretized spiral jet mill PBM, and showed that the convolution between classification and breakage in the jet mill led to non-identifiable parameters in the breakage kernels.

Computational fluid dynamics (CFD) relies on the numerical solution of the Navier–Stokes equations to resolve the fluid flow field within the mill. The CFD simulations of Kozawa et al. [20] showed that coarse particles near the upper wall could escape the mill easily. Similarly, Rodnianski et al. [15] reported the invariability of the spin number with gas mass flow rates. However, as the operation of the jet mill involves a complex interplay between the fluid and particle phases, simulations solely via CFD can be misleading. When the influence of particle phase cannot be ignored completely, a coupled CFD—Discrete Element Method (DEM) approach needs to be adopted. The DEM approach solves the Newtonian kinematic equations for individual particles to determine their trajectories. Given the computational limitations, the CFD-DEM approach cannot be used for very fine particles undergoing breakage. Thus, its application to modelling spiral jet mills requires simplifying assumptions.

Han et al. [24] reported the influence of the feed rate, feed nozzle angle, and the gas flow rate on the product particle size based on two dimensional CFD-DEM simulations. Levy and Kalman [21] presented three-dimensional simulations of particle motion in an industrial scale jet mill. Although particle breakage and particle–particle interactions are completely ignored, the simulations provide interesting insights into the flow field in the jet mill. Teng et al. [12] included particle–particle interactions but ignored particle breakage. With simulations involving only 1000 particles, they reported the influence of the GP on the particle velocity distribution. Along with an increase in the particle velocity, increasing the GP also led to an increased width of the particle velocity distribution.

Moreover, particle–particle collisions were shown to be the primary cause of breakage. By identifying the collision patterns, they concluded that the majority of collisions had a much larger tangential component, which would lead to abrasion rather than fragmentation. Brosh et al. [25] adopted the breakage model developed by Kalman et al. [26] to incorporate comminution in the simulations. To avoid an excessive number of particles in the simulation, particles that fell below 10 µm were removed from the simulation.

Bnà et al. [27] presented a thorough CFD-DEM simulation study. The cut size determined by their simulations is in good agreement with the previous studies by Dobson and Rothwell [4]. They also highlighted the influence of the product hold up inside the mill. They concluded that the fluid deceleration caused by the presence of the particle phase was responsible for the classification efficiency of the mill. However, only a one-way coupling between CFD-DEM was used. In such a coupling approach, the effect of the fluid phase on the particulate phase was considered; however, the effect of the particulate phase on the fluid was ignored.

Given the importance of hold up, Bnà et al. [27] recognized this limitation and underlined the need for a four-way coupling between CFD and DEM. Scott et al. [28] presented such a simulation study using a four-way CFD-DEM coupling. Their simulation reported a decrease in the tangential velocity component with increasing hold up. They also showed that most energy was dissipated along the bed surface and in front of each jet.

Although the use of CFD-DEM models for predictive purposes is limited by computational restrictions, they provide useful insight into the particle dynamics of a mill. In this paper, a coupled CFD-DEM simulation is used to analyse the particle dynamics in the spiral jet mill. Unlike the coupling used by Bnà et al. [27], the coupling used here considers both the influence of fluid on particles and particles on fluid. In the subsequent sections, the CFD-DEM approach is described, followed by the simulation results and conclusions.

## 2. Numerical Methods

**Figure 1:**
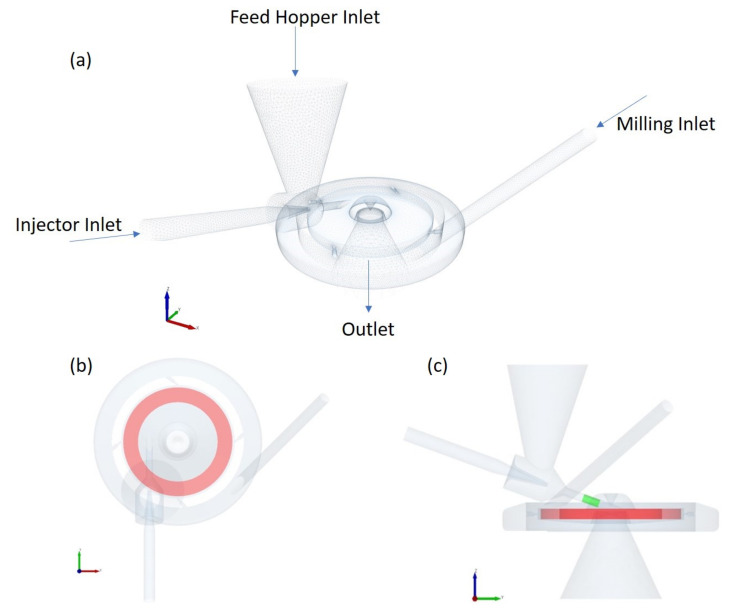
The isometric view (**a**) of the CAD geometry used for CFD-DEM simulations of the jet mill [28,29]. The red section in the top (**b**) and front (**c**) view of the geometry depicts the particle factory in which the initial particle bed is generated. The green section in the front view (**c**) depicts the particle factory through which particles are dynamically fed once the initial particle bed is dispersed.

The mill geometry used for the simulations is based on the Hosokawa AS50 spiral jet mill. However, the geometry is based on an in-house drawing made at the University of Leeds and shown in Figure 1 [28,29]. The milling chamber is 50 mm in diameter and has four jets angled at $50^{\circ }$ from the radius. A special feature of the AS50 is its classifier design. The milling gas spirals up into the classifier section where a vortex finder reverses the flow direction. Figure 1a shows the CAD geometry of the mill used. Following Dogbe [29], an annular manifold for gas distribution was included around the milling chamber as this influences the fluid flow field within the chamber.

The numerical simulation of the process proceeds in three stages. The fluid field is resolved by CFD (using ANSYS Fluent v19), the particle phase is resolved by DEM (using EDEM 2019), and the coupling responsible for exchanging information on solid–fluid forces is achieved by EDEM’s coupling tool. For the fluid field resolution, the gas is assumed to behave ideally. The *k*-ω shear stress transport (SST) [30] was used as the turbulence model. For its simplicity, the Morsi–Alexander correlation [31] was used to compute the fluid drag on the particles.

There are several other drag laws available in the literature, and the choice influences the results of the simulation. Although an evaluation of drag laws is out of the scope of this paper, a few other studies have made the comparisons in the context of coupled CFD-DEM [32,33,34]. The CFD simulations were carried out using the commercial software—ANSYS Fluent v19 with the first-order upwind approach used to solve the individual equations. The convergence tolerances were set at $10^{-4}$ for all the equations. All the boundary conditions were set to pressure type boundaries. For the ‘feed hopper inlet’ and the ‘mill outlet’, atmospheric conditions were assumed.

The ‘injector inlet’ and ‘milling inlet’ were set at the desired operational conditions. Two operating conditions were considered: 1 bar IP and GP, and 3 bar IP and GP (based on gauge pressure). A tetrahedral mesh was used with its size determined by the particle size was used in the DEM simulation. The mesh size was constrained to be 40% larger than the particle diameter [35]. A mesh convergence study was performed by recording the velocity gradient across the milling chamber. Following Norouzi et al. [35], the time step for the CFD simulations was set to 50 times the DEM time step (i.e., at $1\times 10^{-5}$ s).

For the DEM simulations, the particles were considered to be monosized perfect spheres of 200 µm diameter. The particle properties used in the simulations are listed in Table 1. The CFD-DEM coupling was handled by EDEM’s coupling tool, which is based on the approach described by Tsuji et al. [36]. The mass of particles in the cell was decomposed into a weighted volume so that the pressure calculation could be performed. The respective velocity was then returned to EDEM to update the drag force acting on each individual particle. The Hertz–Midlin model was used to model the contact forces. The integration time step was fixed to 20% of the Rayleigh timestep. For the particle size and particle properties used in the simulation, the time step used was $2\times 10^{-7}$ s.

To evaluate the effect of the hold up, three different particle loadings were considered: 10,000 particles (≈0.06 g), 40,000 particles (≈0.25 g), and 100,000 particles (≈0.63 g). In reality, the particles dropped into the feed hopper inlet are sucked into the milling chamber by the injector gas flow. To avoid extensive simulation times, a particle bed was pre-generated in a ring shape factory placed inside the milling chamber as depicted in red in Figure 1b. Once the particle bed is dispersed by the flow and has reached a steady state, 10,000 particles are fed to the mill via the injector factory (coloured green in Figure 1c) at a feed rate of 1.8 kg/h. It takes about 0.128 s to finish feeding the particles. The particle and collision data were collected for 0.15 s from the start of the feeding phase.

## 3. Results

### 3.1. Fluid and Particle Dynamics

**Figure 2:**
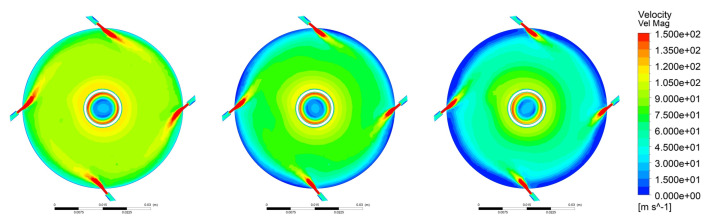
The velocity magnitude contours at the midplane for three particle loadings.

Figure 2 illustrates the velocity magnitude within the milling chamber at the mid plane. These contours are plotted once the pre-generated particle bed is completely dispersed. Although the fluid velocity reaches a magnitude of around 300 m/s, the contour plot is clipped at 150 m/s to emphasize the lower velocity areas. The areas with low fluid velocities are coloured blue, and high fluid velocities are coloured red. Once the pre-generated particle bed is dispersed, particles form a bed on the mill periphery along which they circulate. For all three loadings, the periphery of the mill along which the particles circulate had the lowest velocity.

This particle bed is locally dispersed by the high velocity jet streams emitting from the nozzles. As higher particle loadings lead to a thicker circulating particle bed, the low velocity region around the wall increases in size. Moreover, the jets dispersing the particle bed loose energy much faster when the particle bed is thick. Thus, the length of the jet stream reduces with increasing particle load. This also leads to much lower fluid velocities in the entire mill. This decrease in jet penetration length was also reported by Scott et al. [28] for an even higher particle loading. It is evident that higher particle loadings lead to a larger dampening of the fluid velocity. The velocity magnitude increases radially toward the centre in all cases.

**Figure 3:**
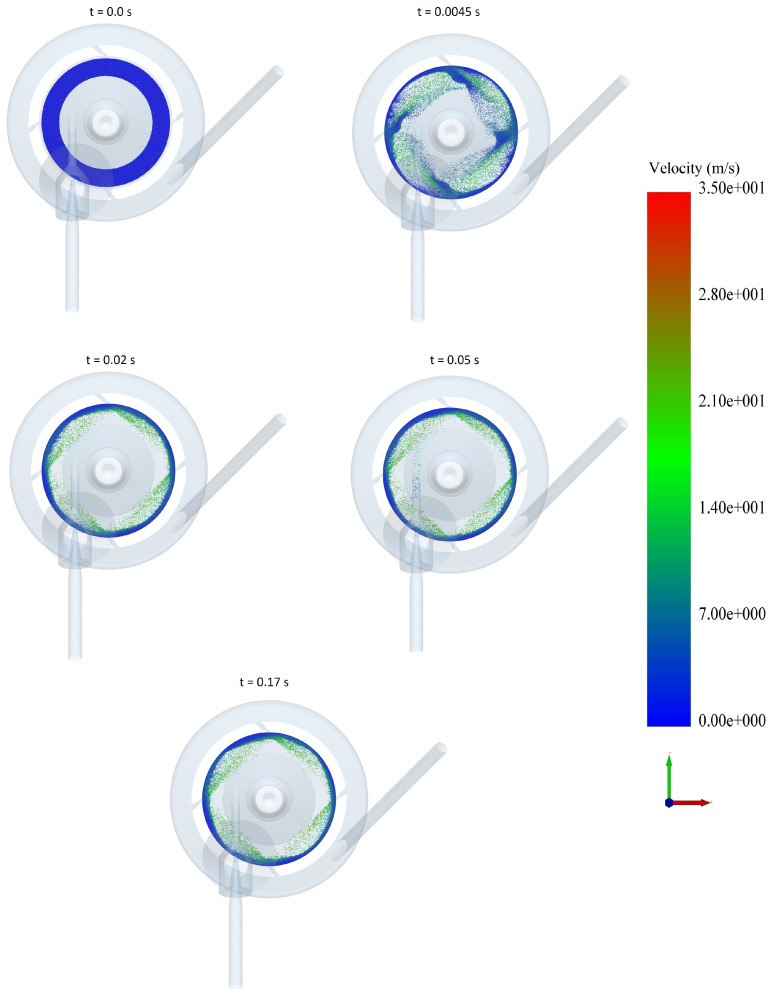
Particle motion in the jet mill for 100,000 particles and 3 bar pressure.

Snapshots of particle motion in the jet mill for the case with 100,000 particles and 3 bar pressure are presented in Figure 3. At the time of 0.0 s, the pre-generated particle bed is intact, and the CFD-DEM simulation is started. The particles start dispersing as the large tangential component of the velocity accelerates them towards the wall. A steady state is reached around 0.015 s, and a thick layer of particles is formed at the mill periphery, which moves along the wall at a very low velocity. Meanwhile, the jet streams disperse the particles when they approach the nozzles. Once the steady state has been achieved, 10,000 new particles are fed through a factory created in the injector nozzle at rate of 1.8 kg/h, taking roughly 0.128 s.

**Figure 4:**
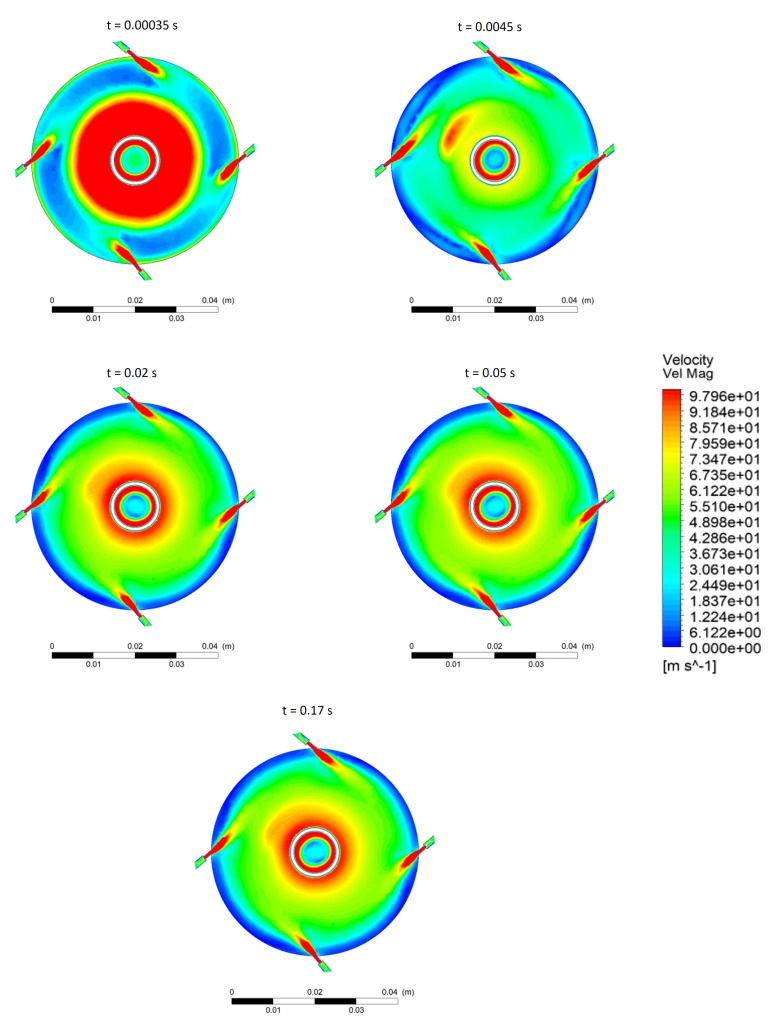
Evolution of fluid field in the jet mill along the mid plane for 100,000 particles with an operating pressure of 3 bar.

The evolution of the fluid field as the particles disperse is illustrated in Figure 4. As can be deduced from the blue ring in the contour plot at time 0.00035 s, the pre-generated particle bed slows the fluid notably. The particle bed, shown by the blue area, is pushed to the wall as it is dispersed. Once the steady state is reached, the velocity profile does not vary greatly. Even when new particles are fed, the velocity profile stays consistent. Thus, it can be said that the particles being entrained into the milling chamber from the feeder do not affect the fluid field in the mill. However, over time, as the particles build up in the mill, the fluid behaviour will be affected. This is evident from Figure 2. The presence of a particle bed circulating along the wall periphery has been reported in previous experimental and numerical studies [6,12,28].

**Figure 5:**
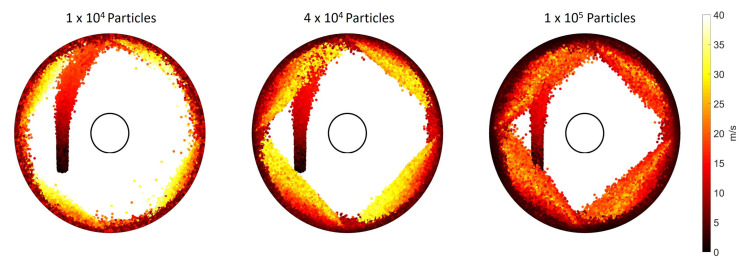
Particle velocity heat map for 0.15 s from the start of the feeding.

Figure 5 displays heat maps of the particle velocities. The figure maps every particle for 0.15 s from the time particle feeding starts. All the particles are coloured according to their velocity at the given time. The prominence of the slow moving particle bed on the mill periphery is evident for all three particle loadings. Similar to the observations from the fluid fields, the increase in the thickness of this bed with particle loading is clear. In all cases, particles with the highest velocity lie along the jet trajectory.

**Figure 6:**
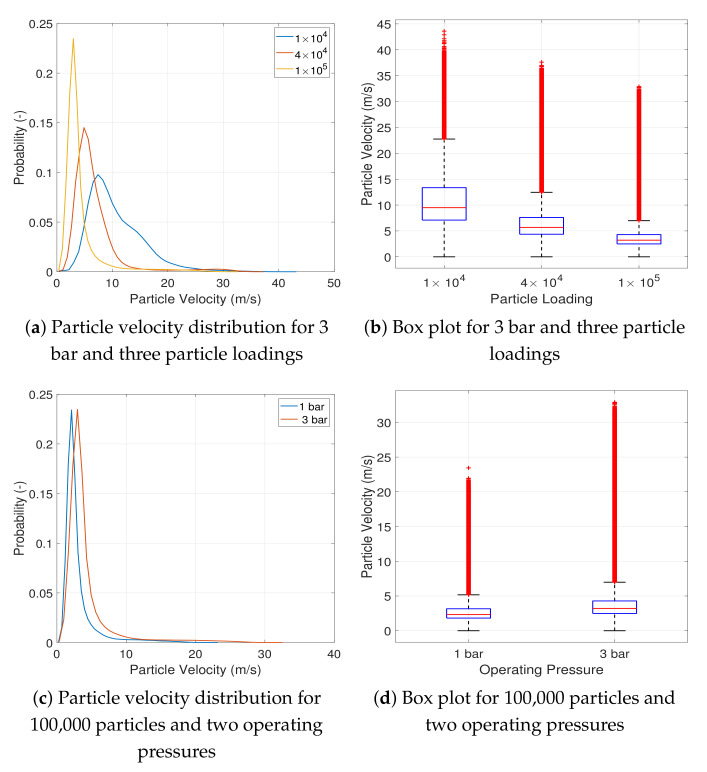
Probability distribution and box plot for particle velocity with increasing particle loading and two operating pressures.

Figure 6a,b illustrate the effect of particle loading on the particle velocity distribution. For both operating pressures, the particle velocity distribution shifts to the left with increasing particle loading. The average and maximum particle velocities also decrease with increasing particle loading. At low particle loadings, the mean free path (distance travelled without colliding) of a particle is much larger. Thus, the particle can be accelerated over a larger distance. Moreover, more fluid kinetic energy per unit particle mass is available for the acceleration.

The effect of operating pressure can be discerned from Figure 6c,d wherein the particle velocity distribution and box plot are depicted for 100,000 particles at the two operating pressures. Increasing the operating pressure leads to a slight rightwards shift in the particle velocity distribution while a much larger increase is noticed in the average and maximum particle velocity. The increase in the velocity distribution is due to the much higher kinetic energy provided by the fluid at higher operating pressures.

**Figure 7:**
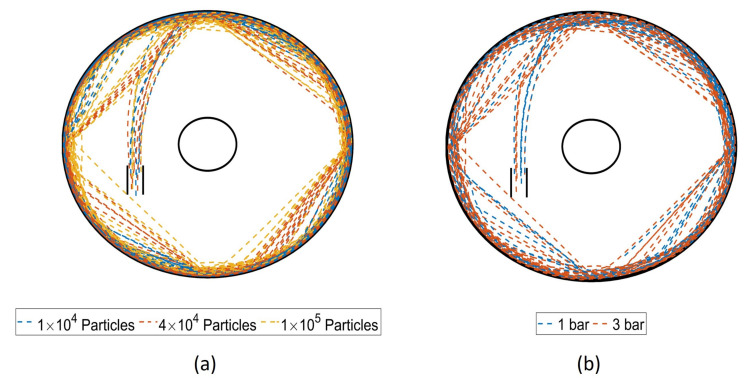
Streamlines of first five particles fed to the mill while describing their motion in the mill for 0.15 s after the feeding. Case (**a**) depicts the streamlines for three particle loadings at 3 bar operating pressure, and case (**b**) depicts the streamlines at two operating conditions and particle loading of 100,000 particles.

The motion of the particles fed into the jet mill via the injector is illustrated by the streamlines plotted in Figure 7. The image on the left depicts the three particle loadings at 3 bar operating pressure, and the image on the right depicts the streamlines for the two operating pressures with 100,000 particles. With higher particle loading, the particles are ejected from the bed by the jet stream move more towards the centre of the mill. Similarly, the particles travel closer to the classifier at higher operating pressure. In all cases, no particles escape the mill via the classifier.

As Bnà et al. [27] indicated, particle classification is a function of the fluid deceleration caused by the particle phase. At the current particle concentrations, very small particles could be entrained. The cut size can be derived by a force balance between the centrifugal and radial forces [4,14,15,16]. However, the particle size used in the current study is much larger. Thus, no classification was observed from the simulations. Even with around 250,000 particles (some as small as 160 µm), Scott et al. [28] could not observe classification in their four-way coupled simulations. This highlights the importance of the solid hold up within the mill on the final particle size of the milled and classified product.

### 3.2. Particle Collision Statistics

Breakage in jet mills occurs when the particles accelerated by the fluid collide with other particles or the wall. If the dissipated energy due to the collisions is higher than a threshold, then the particle breaks. Particles that do not break may experience significant weakening. Previous studies proposed the use of a “fatigue function” to account for this weakening [26,38]. Although this study does not explicitly consider particle breakage, this section presents some results on the collision statistics of particles in the jet mill for all the case studies. All the collision statistics were collected for a time of 0.15 s after the feeding of 10,000 particles begins.

**Figure 8:**
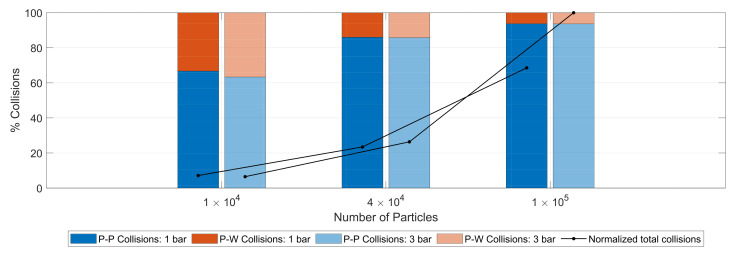
The number of particle–particle (P–P) and particle–wall (P–W) collisions as a percentage of the total collisions for three loadings and two operating pressures. The total number of collisions as a normalized percent of the maximum collisions across all conditions. Collision data collected over 0.15 s after the start of particle feeding.

In Figure 8, the particle–particle and particle–wall collisions are presented as a percentage of the total number of collisions for the given case. The black dots (connected by the solid black lines) lying on the bars corresponding to the simulation cases represent the total number of collisions for those cases as a percentage of the maximum number of collisions observed across all the case studies. In general, increased particle loading led to an increase in the number of collisions.

In all the cases considered, particle–particle collisions were prevalent. At low particle loading, a significant fraction of the collisions occurred between the particle and the wall. The effect of the operating pressure on the number of collisions was prevalent at high loadings. For 100,000 particles, 1 bar operating pressure led to only around 60% of the collisions as 3 bar pressure. For 10,000 particles, increased pressure also led to a slight increase in the fraction of particle–wall collisions.

**Figure 9:**
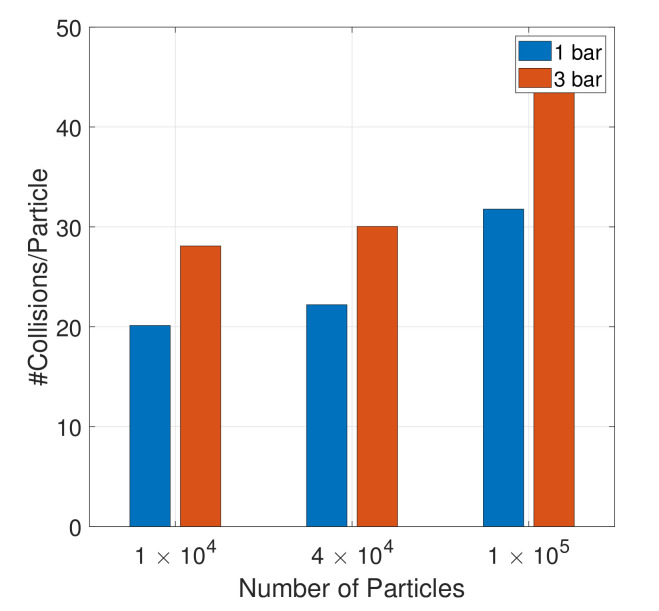
The total number of collisions per particle for the three loadings and two operating pressures over 0.15 s after the start of the feeding phase.

Figure 9 reports the number of collisions per particle for increasing particle loading and the two operating pressures. Again, increased pressure and increased loading both led to an increase in the number of collisions experienced by a particle.

**Figure 10:**
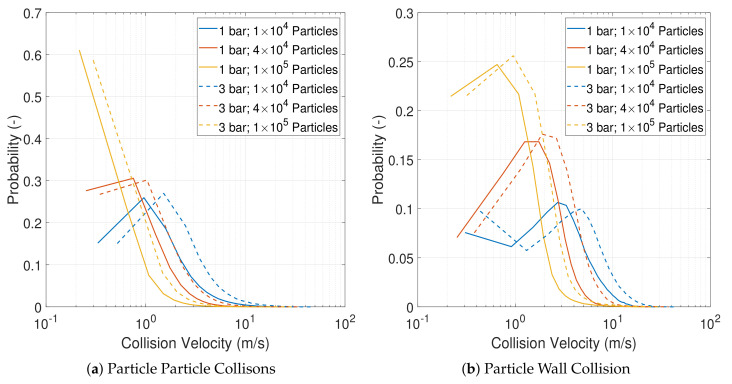
Collision velocity distribution for particle–particle (P–P) and particle–wall (P–W) collision for all cases. Collision data collected over 0.15 s after the start of particle feeding.

The distribution of collision velocities is illustrated in Figure 10. For both particle–particle and particle–wall collisions, increasing particle load leads to a reduction in the impact velocities. This can be explained by the fact that, at lower particle concentrations, each particle travels on a longer mean free path, thus, accelerating to higher velocities before the collision. The high impact collisions at low loading and high operating pressure will lead to a higher degree of breakage. This is corroborated by experimental studies that showed that finer product was obtained at low feed rates and high operating pressures [7,8,11,12].

As the study by Bnà et al. [27] involved only a one-way coupling in which particles mostly followed the fluid streamlines, they reported particle–wall collision velocities of around 40–100 m/s. These were much higher than the collision velocities observed in this study. Moreover, in contrast to the findings in this study, they reported no significant effect of particle loading on the particle–wall collision velocity distribution. This shows the importance of the fluid deceleration by particle phase for breakage as well as classification.

**Figure 11:**
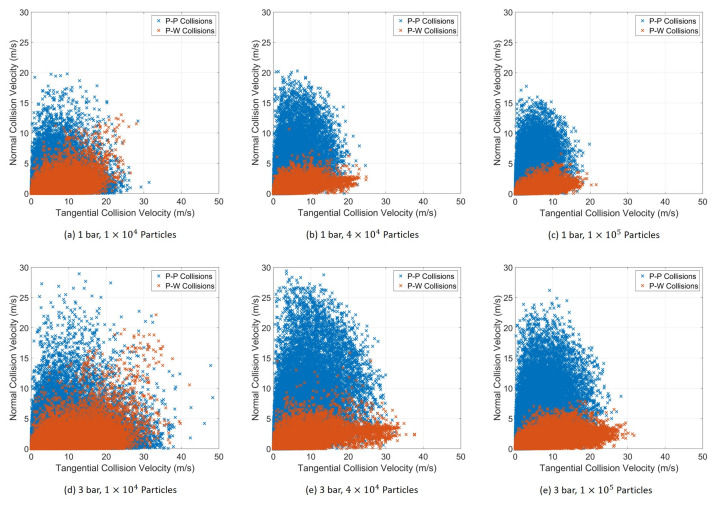
Scatter of the tangential component and normal component of the collision velocity for all cases studied. Collision data collected over 0.15 s after the start of particle feeding.

Figure 11 shows the scatter plots of the tangential component against the normal component of the particle–particle and particle wall collision velocities. As already mentioned, in all cases, the particle–particle collisions are prevalent. Increasing the particle loading led to a decrease in the velocity magnitudes of both the components while increasing the operating pressure led to an increase. At higher particle loadings, the particle–wall collisions were characterized by a high tangential component, while the particle–particle collisions had a much higher normal component.

This contradicts the findings of both Bnà et al. [27] and Teng et al. [12]. Both studies reported a higher tangential component for all collisions. As mentioned, the study by Bnà et al. [27] used only a one-way coupling scheme and, thus, ignored the fluid deceleration by the particle phase. The study by Teng et al. [12], although a two-way coupling, only considered 1000 particles. Such a low particle concentration cannot decelerate fluid to the extent observed in the current study.

## 4. Conclusions

We analysed the effect of particle loading and the operating pressure on the fluid and particle dynamics in a spiral jet mill through coupled CFD-DEM simulations. Three particle loadings and two operating pressures were considered in the study.

We found that the particles significantly decelerated the fluid. The particles dispersed from the pre-generated bed formed a new bed along the mill periphery along which they moved at very slow velocities. The reduction in the fluid velocities was at the maximum in this area. The velocity profile increased monotonically along the radius from the wall toward the mill centre. The particle loading and operating pressure had a much higher impact on the tangential velocity than on the radial velocity. At increasing particle loading and decreasing pressure, the particles experienced more radial forces, which led to the entrainment of coarser particles.

The particle velocity also followed the same trend as the fluid velocity. The particles in the bed moving along the mill periphery were ejected with force by the jet streams. The highest particle velocities were observed directly in front of the jets. Higher operating pressure and low loading led to higher particle velocities and, subsequently, higher impact velocities. In all cases, substantially more particle–particle collisions were reported compared with particle–wall collisions. Most high impact collisions occurred at the surface of the circulating particle bed.

The results show that the particle loading had a profound effect on the fluid field, which, in turn, influenced both the breakage and classification. The computational method and the results presented provide a valuable tool-process optimisation for industrial applications of spiral jet mills. 

## Figures and Tables

**Table 1:** The particle properties used in the DEM simulations [28,29,37].

Particle Property	Value
Diameter	200 m
Density	1525 $\mathrm{kg}/m^{3}$
Young’s Modulus	2.7 × 10${}^{8}$ Pa
Poisson’s Ratio	0.35
Coefficient of Restitution	0.5
Coefficient of Static Friction	0.5
Coefficient of Rolling Friction	0.01
